# Association of glycemic control and chronic kidney disease with hospitalization in type 2 diabetes in a cross-sectional study in Region Halland

**DOI:** 10.1080/02813432.2025.2591340

**Published:** 2025-12-01

**Authors:** Bertin Magamba, Junmei Miao Jonasson, Björn Agvall

**Affiliations:** a Vårdcentralen Falkenberg, Falkenberg, Sweden; b School of Public Health and Community Medicine, Institute of Medicine, Sahlgrenska Academy, University of Gothenburg, Gothenburg, Sweden; c Department of Research and Development, Region Halland, Halmstad, Sweden; d Department of Clinical Sciences, Center for Primary Health Care Research, Malmö, Lund University, Malmö, Sweden

**Keywords:** Type 2 diabetes, chronic kidney disease, healthcare utilization, hospitalization, glycated hemoglobin A, blood glucose control

## Abstract

**Aims:**

To examine the separate associations of glycemic control and chronic kidney disease with hospitalization days over one year in patients with type 2 diabetes (T2D).

**Materials and methods:**

A cross-sectional study using retrospective data on T2D patients in Region Halland, Sweden, during 2020. Data from the Region Halland database included hospitalizations, emergency visits, primary care encounters, glycemic control, estimated glomerular filtration rate (eGFR), comorbidities, pharmacotherapy and healthcare encounters. Negative binomial regression was used to assess associations with healthcare utilization.

**Results:**

A total of 12,689 patients participated, with an average age of 66.0 years (67.1 years for women and 65.2 years for men, *p* = 0.010). Higher glycated hemoglobin (HbA1c) and elevated blood glucose levels were associated with increased hospitalization days. Relative risks (RRs) for hospitalization days were higher for HbA1c 52–70 mmol/mol (RR 1.08, 95% CI 1.01–1.15) and >70 mmol/mol (RR 1.24, 95% CI 1.12–1.37). Elevated blood glucose levels ≥7.8 mmol/L had an RR of 1.17 (95% CI 1.04–1.32), while <7.8 mmol/L had an RR of 0.85 (95% CI 0.76–0.96). Patients with an eGFR between 30 and 60 mL/min had a RR of hospitalization of 1.35 (95% CI 1.26–1.44) compared to those with an eGFR >60 mL/min. For patients with an eGFR <30 mL/min, the RR of hospitalization was 3.36 (95% CI 3.00–3.77).

**Conclusions:**

Both poor glycemic control and decreased kidney function were associated with higher hospitalization rates in T2D patients. These findings emphasize the need for effective management of glycemic control and renal function to reduce the healthcare burden in this patient population.

## Introduction

The prevalence of type 2 diabetes (T2D) in Nordic countries has been rising significantly over recent years. In Sweden, the prevalence of T2D treated with glucose-lowering drugs increased by 61% from 2006 to 2013, reaching 4.4% in 2013 [1]. More recent estimates indicate that the age-standardized prevalence of diabetes among adults aged 20–79 in high-income countries, including Sweden, was 10.2% in 2024 [2]. Additionally, a population-based study in one city – Malmö showed that the prevalence of T2D doubled between 2011 and 2018, increasing from 2.46% to 4.26% [3]. In Denmark, the prevalence of T2D was 4.4% in 2016, with annual increases of 5.5% [4]. These trends underscore the growing public health challenge posed by T2D.

The increasing prevalence of T2D is driven by environmental and lifestyle factors, as well as aging populations [5]. T2D substantially impairs health-related quality of life, with complications such as obesity, cardiovascular disease and hypoglycemic episodes further exacerbating its burden [6]. T2D is associated with various complications and comorbidities, particularly cardiovascular diseases, affecting 28% of patients in Sweden [7–9]. Complications are typically classified as microvascular (retinopathy, nephropathy, neuropathy) or macrovascular, the latter resulting from atherosclerosis and potentially leading to myocardial infarction, stroke, angina and peripheral vascular disease [5,10,11]. The risk of these complications increases significantly with long-standing T2D and poor glycemic control [10].

Excess mortality in individuals with T2D has been associated with poor glycemic control and renal complications, particularly in relation to cardiovascular and all-cause mortality [12–14]. Data from the Swedish National Diabetes Register (NDR) revealed that over a 4.5-year period, 18% of individuals with T2D died compared to 15% of age-, sex- and county-matched controls from the general population, while cardiovascular mortality was 8% among patients versus 6% in controls [15]. Notably, mortality risks were higher in younger individuals, particularly those with poor glycemic control and severe renal complications. In contrast, older patients with good glycemic control experienced lower mortality risks suggesting the importance of regulating glucose levels.

The burden of T2D extends to healthcare systems and the economy, with costs largely driven by its complications [16–18]. Poor glycemic control is associated with increased healthcare utilization, including more frequent hospitalizations and outpatient visits [19,20]. T2D in combination with chronic kidney disease (CKD) have indicated worse outcomes. Treatments with SGLT-2 inhibitors and GLP-1 have positive influence on both glucose regulation and kidney function. Individuals with T2D are typically followed primarily for diabetes-related management within primary care in Sweden and probably other Nordic countries. The presence of cardiovascular disease further intensifies resource use, as patients with both T2D and cardiovascular disease require significantly more care than those without [17]. Hospital admission rates are influenced by factors such as bed availability, continuity of care, and characteristics of primary care, including access, task profiles and medical equipment [21].

The aim was to investigate whether suboptimal glycemic control and different stages of CKD were associated with the number of hospitalization days over a one-year period among individuals with T2D.

## Materials and methods

The present study was a cross-sectional study based on retrospective data on patients with T2D in Region Halland during the study period from 1 January 2020 to 31 December 2020. Located in the southwest of Sweden, Region Halland had an estimated population of 330,000 inhabitants. The region included three acute care hospitals, 40 inpatient wards, two emergency departments, 30 specialized outpatient clinics and 48 primary care facilities.

### Data source

The collection of patient data was facilitated through the utilization of the Regional Healthcare Information Platform (RHIP), a platform that houses comprehensive electronic healthcare records of residents within Region Halland [22]. This platform encompassed data from both hospital care and primary care services provided by public and private healthcare providers. RHIP contained a wide range of information, including sociodemographic details of the patients, diagnoses based on the International Classification of Diseases (ICD), complete clinical laboratory assessments, radiological examination results and healthcare delivery resources. Two sources within RHIP were utilized to acquire data related to pharmacotherapy: the Swedish Prescribed Drugs Register and the pharmacy’s dose dispensing unit known as Apodos. These sources provided the necessary information on medication prescriptions and dispensing details.

### Study population

The study enrolled individuals aged >30 years who had been diagnosed with T2D based on the ICD codes E11–E14 during the lookback period from 2013 until the end of 2019. Individuals with a new diagnosis of diabetes during the study period of 2020 were excluded. To be included in the study cohort, patients needed to meet the inclusion criteria of being residents of Region Halland and receiving healthcare within the same region. Patients newly diagnosed with T2D in 2020 were excluded to ensure that the study cohort consisted of individuals with established diabetes, allowing for the evaluation of long-term disease management and healthcare utilization patterns. This exclusion criterion minimizes potential confounding effects related to initial treatment adjustments and newly diagnosed cases with limited follow-up data. Flowchart for the study is presented in Supplementary Figure 1.

### Data collection

Age was defined based on each patient’s age as of 1 January 2020, to maintain consistency across all analyses. While this approach provides a standardized reference point, we acknowledge that it may not fully account for within-year aging effects. Participants ≤30 years were excluded to restrict analyses to an adult T2D population, improve cohort homogeneity and limit confounding from atypical diabetes etiologies. Data regarding sex and comorbidities were collected. Comorbidities were identified based on ICD codes recorded between 2013 and 2019 to capture a comprehensive history of chronic conditions. While some conditions may have changed by 2020, this approach ensures that pre-existing disease burden is accounted for in the analysis. The comorbidities included ischemic heart disease (ICD codes I20–I29), hypertension (I10), heart failure (I50, I42), peripheral arterial disease (I73.9), cerebrovascular disease (I63–I69), chronic obstructive pulmonary disease (J44) and diabetic retinopathy, which was recorded using ICD codes H36 and E113.

Laboratory data from 2020 were averaged for the year and included blood values regarding kidney function measured as estimated glomerular filtration rate (eGFR), B-glucose, glycated hemoglobin (HbA1c) and lipid profiles (total cholesterol and LDL cholesterol). Due to variation in timing and context, all B-glucose values were treated as non-fasting. These measurements were primarily used for monitoring glycemic regulation rather than for diagnostic purposes. Systolic blood pressure values were included within the range of 80–200 mmHg. Measurements outside this range were excluded as potential outliers, as values below 80 mmHg may reflect acute hypotensive episodes rather than chronic blood pressure status, while values above 200 mmHg may indicate measurement errors or transient hypertensive crises rather than sustained hypertension. This range is consistent with previous studies analyzing blood pressure distributions in patients with T2D and aligns with clinical plausibility thresholds.

Medication data were registered based on the drugs dispensed to patients, as recorded in the pharmacy records for the years 2017 and 2020. Five drug classes were analyzed using their Anatomical Therapeutic Chemical (ATC) codes: diabetes pharmacotherapies (A10), diuretics (C03), beta-blockers (C07), renin–angiotensin system inhibitors RASi (C09), calcium antagonists (C08) and statins (C10).

Healthcare utilization in the year 2020 was recorded, including hospital admissions and total hospital days occurring between 1 January 2020 and 31 December 2020. This measure includes all hospitalizations and rehospitalizations with a primary diagnosis coded under ICD-10 I-codes (cardiovascular disease) or E-codes (endocrinological conditions, which was mainly diabetes mellitus E11–E14). Individuals who died during the study year were included in the analysis. Their healthcare utilization, including hospital days, was recorded up to the date of death. Hospitalizations outside these diagnostic categories were excluded. The healthcare variables covered inpatient care, which included hospital admissions due to CVD and hospital bed days, emergency department encounters, and hospital-related outpatient care. This outpatient care involved visits to physicians, registered nurses and paramedical professionals such as dietitians, physiotherapists, occupational therapists and psychologists. Primary healthcare visits were recorded, including encounters with physicians, specialist nurses, registered nurses and paramedical professionals such as dietitians, physiotherapists, occupational therapists and psychologists. Outpatient visits, including emergency department encounters, physician consultations, and nurse or paramedical visits, were recorded as individual events. These were analyzed separately from inpatient care, which was measured in total hospital days.

### Statistical analysis

Descriptive statistics were performed to summarize the baseline characteristics of the study population. Continuous variables, such as age, blood pressure and laboratory values, were expressed as means and standard deviations (SDs). Group comparisons of continuous variables were performed using Student’s *t*-test for two groups or the Kruskal–Wallis test when comparing more than two groups, as the data were not normally distributed. Categorical variables, such as gender, medication use and disease classifications, were presented as frequencies and percentages. Comparisons between categorical variables were conducted using the Chi-square test. For all variables, the number and percentage of missing data were reported.

Other variables, such as HbA1c, total cholesterol, LDL cholesterol, kidney function and blood pressure, were grouped into clinically relevant categories for analysis. HbA1c was classified into <52 mmol/mol, 52–70 mmol/mol and >70 mmol/mol and those having only a B-glucose were categorized to B-glucose <7.8 mmol/L and ≥7.8 mmol/L [23]. The inclusion of both HbA1c and blood glucose in the same category was done to account for patients who had only blood glucose measurements available. B-glucose categorization was applied only to patients without HbA1c data. For patients with HbA1c measurements, B-glucose values were not included in the analysis to avoid overlapping glycemic indicators. Total cholesterol was categorized as normal or elevated (>4.5 mmol/L), and LDL cholesterol was grouped similarly with an elevated threshold of >2.5 mmol/L. Kidney function was classified with eGFR into three categories, with values >60 mL/min/1.73 m^2^, between 30 and 60 mL/min/1.73 m^2^ and <30 mL/min/1.73 m^2^ [24,25]. Systolic blood pressures were defined as normal when <130 mmHg and elevated when ≥130 mmHg [26,27]. Imputation was not performed due to the low percentage of missing data and its minimal impact on the analysis, as the data appears to be missing at random. Spearman’s rank correlation coefficient was used to investigate the correlation between number of hospital days and primary care encounters. This test was chosen as it does not require the assumption of normal distribution. Negative binomial regression was used to analyze factors associated with hospital days due to the high variability and skewed distribution of the data. The analysis included two models, i.e. crude model (model 1) and adjusted model (model 2) which included adjustment for age, sex, glycemic control, kidney function, comorbidities and pharmacotherapy. The results are presented as relative risks (RRs) with 95% confidence intervals (CIs). A *p* value of less than 0.05 was considered statistically significant. All statistical analyses were performed using IBM SPSS Statistics version 30 (IBM Corp., Armonk, NY).

## Results

A total of 12,689 patients were included in the study. The average age of the cohort was 66.0 years, with 7472 men, representing 59% of the total. Women had an average age of 67.1 years, while men had an average age of 65.2 years (*p* = 0.01). An overview of the basic characteristics of the participants is presented in Table 1.

**Table 1.** Basic characteristics of the study cohort, presented in total and by HbA1c and blood glucose levels.

	Glycemic measurements					Total	*p* Value
	HbA1c <52 mmol/mol	HbA1c 52–70 mmol/mol	HbA1c >70 mmol/mol	B-Glucose <7.8 mmol/L	B-Glucose ≥7.8 mmol/L		
Total cohort	4883 (38)	4556 (36)	1212 (10)	1148 (9)	890 (7)	12,689 (100)	
*Age*							
Age, means (SD)	66.0 (10.9)	66.0 (10.9)	64.3 (12.3)	66.8 (10.7)	66.6 (10.9)	66.0 (11.0)	<0.001
*Sex, n (%)*							
Women	2058 (42)	1852 (41)	497 (41)	510 (41)	300 (34)	5217 (41)	<0.001
Men, *n* (%)	2825 (58)	2704 (59)	715 (59)	638 (57)	590 (66)	7472 (59)	
*Comorbidities*							
Hypertension, *n* (%)	2312 (47)	1765 (39)	426 (35)	595 (52)	393 (44)	5491 (43)	<0.001
ASCVD, *n* (%)	892 (18)	621 (14)	182 (15)	192 (17)	117 (17)	2004 (16)	<0.001
IHD, *n* (%)	608 (13)	467 (10)	119 (10)	135 (12)	77 (9)	1406 (11)	<0.001
AMI, *n* (%)	167 (3)	66 (1)	14 (1)	33 (3)	7 (1)	287 (3)	<0.001
Cerebrovascular insult, *n* (%)	224 (5)	120 (3)	42 (4)	42 (4)	26 (3)	454 (4)	<0.001
PAD, *n* (%)	36 (1)	18 (<1)	7 (1)	10 (1)	8 (1)	79 (1)	0.129
HF, *n* (%)	199 (4)	122 (3)	41 (3)	39 (3)	23 (3)	424 (3)	<0.001
COPD, *n* (%)	175 (4)	105 (2)	21 (2)	37 (3)	18 (2)	356 (3)	<0.001

HbA1c: glycated hemoglobin; B-glucose: blood glucose; *n*: number; SD: standard deviation; ASCVD: atherosclerotic cardiovascular disease; IHD: ischemic heart disease; AMI: acute myocardial infarction; PAD: peripheral arterial disease; HF: heart failure; COPD: chronic obstructive pulmonary disease.

A total of 483 (4%) deaths were recorded during the study period. Among those with HbA1c <52 mmol/mol, 155 (3%) deaths occurred, while 179 (4%) deaths were observed in the HbA1c 52–70 mmol/mol group. In the HbA1c >70 mmol/mol group, 70 (5%) deaths were recorded, and for patients with only blood glucose measurements, 79 (4%) deaths occurred (*p* < 0.001).

A total of 2038 individuals (16%) did not have HbA1c measurements but only blood glucose levels available. Among individuals grouped by HbA1c categories, the mean B-glucose levels were 7.5 mmol/L (2.0) in the HbA1c <52 mmol/mol group, 9.2 mmol/L (2.7) in the HbA1c 52–70 mmol/mol group, and 12.1 mmol/L (3.9) in the HbA1c >70 mmol/mol group. For those with only B-glucose measurements, individuals with B-glucose levels <7.8 mmol/L had an average of 6.8 mmol/L (1.0), while those with B-glucose levels ≥7.8 mmol/L had an average of 11.2 mmol/L (3.2). The mean B-glucose level for the entire study cohort was 8.7 mmol/L (3.0), with a statistically significant difference observed across the groups (*p* < 0.001). Laboratory findings and clinical measurements during the study period are presented in Table 2.

**Table 2.** Laboratory findings and clinical measurements during the 2020 study period, presented in total and by HbA1c and blood glucose levels.

	Glycemic measurements					Total	*p* Value
	HbA1c <52 mmol/mol	HbA1c 52–70 mmol/mol	HbA1c >70 mmol/mol	B-Glucose <7.8 mmol/L	B-Glucose ≥7.8 mmol/L		
Total cohort	4883 (38)	4556 (36)	1212 (10)	1148 (9)	890 (7)	12,689 (100)	
*Laboratory findings*							
Kidney function							
eGFR, mean (SD)	67.1 (17.8)	64.8 (19.1)	63.46 (21.5)	66.2 (17.6)	64.2 (18.6)	65.7 (18.7)	<0.001
eGFR >60 mL/min, *n* (%)	2908 (60)	2475 (54)	629 (52)	612 (53)	440 (49)	7064 (56)	<0.001
eGFR 30–60 mL/min, *n* (%)	1215 (25)	1295 (28)	389 (32)	282 (25)	258 (29)	3439 (27)	
eGFR <30 mL/min, *n* (%)	166 (3)	214 (3)	92 (8)	38 (3)	34 (4)	544 (4)	
Missing eGFR	594 (12)	572 (13)	102 (8)	216 (19)	158 (18)	1642 (13)	
B-Glucose							<0.001
B-Glucose mmol/L, mean (SD)	7.5 (2.0)	9.2 (2.7)	12.1 (3.9)	6.8 (1.0)	11.2 (3.2)	8.7 (3.0)	
Cholesterol levels							
P-Cholesterol, mean (SD)	4.3 (1.1)	4.2 (1.1)	4.4 (1.2)	4.4 (1.1)	4.3 (1.1)	4.3 (1.1)	<0.001
P-Cholesterol <4.5 mmol/L, *n* (%)	2808 (58)	2761 (61)	671 (55)	602 (52)	531 (60)	7373 (58)	<0.001
P-Cholesterol ≥4.5 mmol/L, *n* (%)	1822 (39)	1609 (35)	485 (40)	454 (40)	290 (33)	4660 (37)	
P-Cholesterol missing, *n* (%)	253 (5)	186 (4)	56 (5)	92 (8)	69 (8)	656 (5)	
LDL-C, mean (SD)	2.5 (1.0)	2.5 (0.9)	2.5 (1.0)	2.6 (1.0)	2.5 (1.0)	2.5 (0.9)	<0.001
LDL-C <2.5 mmol/L, *n* (%)	2568 (53)	2570 (56)	617 (51)	544 (47)	471 (53)	6770 (53)	<0.001
LDL-C >2.5 mmol/L, *n* (%)	2134 (44)	1856 (41)	550 (45)	541 (47)	362 (41	5443 (43)	
LDL-C missing, *n* (%)	181 (4)	130 (3)	45 (4)	63 (6)	57 (6)	476 (4)	
Blood pressure							
Systolic BP mmHg, mean (SD)	136.4 (13.5)	136.7 (13.7)	137.2 (14.3)	137.8 (13.4)	137.5 (13.5)	136.8 (13.7)	0.011
<130 mmHg, *n* (%)	1381 (28)	1264 (28)	332 (27)	290 (25)	229 (26)	3496 (28)	0.003
≥130 mmHg, *n* (%)	3410 (70)	3210 (70)	841 (69)	841 (73)	652 (73)	8954 (71)	
Blood pressure missing	92 (2)	82 (2)	39 (3)	17 (2)	9 (1)	239 (2)	
*Pharmacotherapies*							
Diabetes pharmacotherapies							
Diet only	832 (17)	215 (5)	59 (5)	271 (24)	82 (9)	1459 (12)	<0.001
Metformin	3529 (72)	3553 (78)	807 (67)	765 (67)	633 (71)	9287 (73)	<0.001
Sulfonylurea	63 (1)	128 (3)	34 (3)	17 (2)	30 (3)	272 (2)	<0.001
GLP-1	569 (12)	1067 (23)	465 (38)	137 (12)	183 (21)	2421 (19)	<0.001
DPP4	608 (13)	1084 (24)	300 (25)	128 (11)	209 (24)	2329 (18)	<0.001
SGLT-2	533 (11)	1157 (25)	361 (30)	115 (10)	210 (24)	2376 (19)	<0.001
Insulin	492 (10)	1292 (28)	701 (58)	123 (11)	259 (29)	2867 (23)	<0.001
Cardiovascular pharmacotherapies							
RASi	3125 (64)	3010 (66)	739 (61)	738 (64)	565 (64)	8177 (64)	0.021
Betablockers	2186 (45)	2095 (46)	597 (49)	493 (43)	404 (45)	5775 (46)	0.018
Calcium channel blockers	1728 (35)	1658 (36)	399 (33)	419 (37)	354 (40)	4558 (36)	0.020
Diuretics	1210 (25)	1256 (28)	425 (35)	268 (23)	228 (26)	3387 (27)	<0.001
Lipid pharmacotherapies							
Statins	3115 (64)	3194 (70)	810 (67)	697 (61)	570 (64)	8386 (66)	<0.001

HbA1c: glycated hemoglobin; eGFR: estimated glomerular filtration rate; B-glucose: blood glucose; LDL-C: low-density lipoprotein cholesterol; SBP: systolic blood pressure; BP missing: missing data for blood pressure; diet only: dietary treatment without pharmacological interventions; GLP-1: glucagon-like peptide-1 receptor agonists; DPP4: dipeptidyl peptidase-4 inhibitors; SGLT-2: sodium-glucose cotransporter-2 inhibitors; RASi: renin–angiotensin system inhibitors.

The hospital admission rate was 0.2 (0.7), and the total cohort had an average of 0.3 (0.9) encounters at the emergency department during the study period. The highest number of visits occurred among patients with HbA1c >70 mmol/mol, who had an average of 11.0 visits to primary care. Patients with a B-glucose level <7.8 mmol/L had an average of 7.7 visits to primary care. Healthcare utilization throughout the study period is summarized in Table 3. The correlation between hospital days and the number of primary care encounters was 0.207 (*p* < 0.001).

**Table 3.** Healthcare utilization in total and distribution by HbA1c, B-glucose levels and CKD stages during the study period.

	Total	Glycemic measurements					Renal function/CKD stages		
		HbA1c <52 mmol/mol	HbA1c 52–70 mmol/mol	HbA1c >70 mmol/mol	B-Glucose <7.8 mmol/L	B-Glucose ≥7.8 mmol/L	Stages 1–2	Stage 3	Stages 4–5
*Hospital care*									
Inpatient care, mean (SD)									
Inpatient care days	1.0 (0.7)	0.9 (3.8)	1.0 (4.2)	1.6 (5.3)	0.6 (3.2)	1.1 (5.4)***	0.7 (3.3)	1.4 (5.0)	4.8 (9.6)***
Hospital admissions	0.2 (0.7)	0.2 (0.6)	0.2 (0.7)	0.3 (0.9)	0.1 (0.5)	0.2 (0.6)***	0.1 (0.5)	0.3 (0.8)	0.8 (1.4)***
Hospital outpatient care visits, mean (SD)									
ED	0.3 (0.9)	0.3 (1.0)	0.3 (0.9)	0.5 (1.1)	0.3 (0.8)	0.3 (0.7)***	0.3 (0.9)	0.4 (1.0)	0.9 (1.3)***
Physician	0.4 (1.2)	0.4 (1.2)	0.4 (1.1)	0.5 (1.4)	0.3 (1.0)	0.3 (1.1)***	0.3 (1.0)	0.4 (1.2)	1.6 (2.8)***
Nurse	0.6 (5.9)	0.7 (6.8)	0.5 (4.5)	1.0 (9.3)	0.3 (4.3)	0.3 (1.7)***	0.3 (1.4)	0.5 (1.9)	7.1 (26.9)***
*Primary care*									
Primary care visits, mean (SD)									
Physicians	3.3 (3.4)	3.2 (3.4)	3.4 (3.3)	3.9 (4.0)	3.0 (3.2)	3.0 (3.1)***	3.3 (3.3)	3.9 (3.7)	4.8 (5.0)***
Nurses	5.7 (7.3)	5.4 (6.8)	6.0 (7.4)	7.1 (9.2)	4.7 (5.7)	6.0 (8.5)***	5.3 (6.4)	7.0 (8.9)	9.1 (11.2)***

CKD: chronic kidney disease; SD: standard deviation; ED: emergency department.

*** *p* < 0.001.

The negative binomial regression analysis showed that higher HbA1c and elevated blood glucose levels were associated with an increased RR of hospitalization days, as were ASCVD, COPD and reduced kidney function. LDL-cholesterol levels <2.5 mmol/L were associated with a lower RR. Lower systolic blood pressure was associated with a higher RR compared to systolic blood pressure above 130 mmHg. Among pharmacotherapies, metformin was associated with a lower RR, while insulin use was associated with a higher RR. The negative binomial regression analysis of factors associated with hospital days is presented in Table 4.

**Table 4.** Negative binomial regression analysis of factors associated with hospital days.

	Model 1				Model 2			
	RR	95% CI			RR	95% CI		
		Lower	Upper	*p* Value		Lower	Upper	*p* Value
Age					1.01	1.00	1.01	<0.001
Men	Reference				Reference			
Women	0.92	0.89	0.96	<0.001	0.90	0.85	0.95	<0.001
*HbA1c and B-glucose*								
<52 mmol/mol	Reference				Reference			
52–70 mmol/mol	1.91	1.76	2.08	<0.001	1.08	1.01	1.15	0.02
>70 mmol/mol	1.18	1.11	1.25	<0.001	1.24	1.12	1.37	<0.001
B-glucose <7.8 mmol/L	6.54	5.21	8.21	<0.001	0.85	0.76	0.96	0.01
B-glucose ≥7.8 mmol/L	11.84	9.43	14.87	<0.001	1.17	1.04	1.32	0.01
*Comorbidities*								
ASCVD	1.93	1.85	2.06	<0.001	1.32	1.23	1.42	<0.001
COPD	2.21	1.94	2.52	<0.001	1.69	1.47	1.95	<0.001
*Clinical findings*								
CKD stage 1–2, eGFR >60 mL/min	Reference				Reference			
CKD stage 3, eGFR 30–60 mL/min	11.58	9.24	14.53	<0.001	1.35	1.26	1.44	<0.001
CKD stage 4–5, eGFR <30 mL/min	6.05	4.82	7.58	<0.001	3.36	3.00	3.77	<0.001
LDL-cholesterol >2.5 mmol/L	0.60	0.53	0.69	<0.001	0.89	0.83	0.95	<0.001
SBP <130 mmHg	1.19	1.12	1.25	<0.001	0.90	0.84	0.96	<0.001
*Pharmacotherapy*								
Metformin	0.43	0.38	0.42	<0.001	0.79	0.74	0.84	<0.001
Insulin	2.45	2.23	2.56	<0.001	1.40	1.31	1.50	<0.001
Statin	1.17	1.11	1.24	<0.001	0.95	0.89	1.02	0.16

RR: relative risk; CI: confidence interval; ASCVD: atherosclerotic cardiovascular disease; COPD: chronic obstructive pulmonary disease; CKD: chronic kidney disease; LDL: low-density lipoprotein; SBP: systolic blood pressure.

Model 1 includes crude estimates. Model 2 is adjusted for age, sex, glycemic control, kidney function, comorbidities and pharmacotherapy.

## Discussion

This study examined healthcare utilization among patients with T2D in Region Halland, Sweden, with a focus on glycemic control and CKD. The results showed that poorer glycemic control, indicated by higher HbA1c and blood glucose levels, was associated with increased hospitalization rates and more frequent primary care visits. CKD was also associated with higher hospitalization rates, suggesting that impaired kidney function, in combination with poor glycemic control, may increase healthcare utilization. These findings emphasize the critical role of both glycemic control and kidney function in managing T2D patients, highlighting the potential benefits of improved management strategies for reducing hospitalization rates.

The results also confirm previous research indicating that poor glycemic control is a strong predictor of adverse outcomes in T2D [1,2]. The observed excess mortality in patients with HbA1c >70 mmol/mol aligns with studies demonstrating that uncontrolled diabetes leads to an increased risk of both macrovascular and microvascular complications, ultimately contributing to higher morbidity and mortality [3,4]. Interestingly, even moderately elevated HbA1c levels (52–70 mmol/mol) were associated with increased mortality risk compared to well-controlled individuals, reinforcing the clinical importance of striving for optimal glycemic targets [28,29].

Importantly, both poor glycemic control and impaired kidney function were strongly associated with increased healthcare utilization, including hospitalization and primary care visits. Patients with HbA1c levels >70 mmol/mol had significantly higher rates of hospitalizations and primary care encounters compared to those with well-controlled glycemia (<52 mmol/mol). There was a positive but week correlation between the number of hospital days and the number of primary care encounters which probably is due to difficult-to-treat patients requiring generally increased care. This observation is consistent with earlier studies indicating that inadequate glycemic control increases the risk of diabetes-related complications, requiring more intensive medical interventions and prolonged care [13,14]. Individuals with suboptimal metabolic control are more likely to require emergency services, specialist consultations and recurrent hospitalization due to acute complications and exacerbations of comorbidities. The findings of this study highlight associations between glycemic control, CKD and healthcare utilization in patients with T2D. In this cohort, 16% of patients had documented ASCVD, with ischemic heart disease affecting 11% and cerebrovascular disease affecting 4%. These descriptive observations are consistent with previous research that has consistently shown an elevated burden of ASCVD among individuals with T2D [9–11]. These findings reinforce the established relationship between T2D and both macrovascular and microvascular complications, underscoring the role of chronic hyperglycemia in promoting endothelial dysfunction, inflammation and atherogenesis [5,14,15,28,29]. Notably, patients with HbA1c levels ≥70 mmol/mol exhibited a higher prevalence of cardiovascular comorbidities, supporting the notion that poor glycemic control exacerbates cardiovascular risk which is supported in previous research which suggests that inadequate metabolic control may contribute to acute cardiovascular events [1,30]. In this study, lower systolic blood pressure was associated with a higher RR compared to systolic blood pressure above 130 mmHg. This paradoxical finding is likely explained by confounding due to underlying frailty or comorbid conditions in patients with lower blood pressure, reflecting a weaker clinical status rather than a protective effect of higher blood pressure. Nevertheless, in a long-term perspective, elevated systolic blood pressure remains a well-established risk factor for adverse cardiovascular outcomes.

Kidney dysfunction also demonstrated a strong association with both poor glycemic control and higher healthcare utilization. Patients with HbA1c levels ≥70 mmol/mol had a substantially higher prevalence CKD, supporting previous evidence linking prolonged hyperglycemia to diabetic nephropathy [28,31,32]. This association stresses the pathophysiological progression of hyperglycemia-induced renal damage and highlights the potential for simultaneous microvascular complications such as diabetic retinopathy. In this cohort, kidney impairment was associated with increased hospital days, particularly in those with an eGFR <30 mL/min, who experienced more than a threefold increase in hospitalization duration compared to those with normal renal function. These findings support the growing emphasis on early identification and management of renal dysfunction in individuals with diabetes.

Patients with poor glycemic control were more likely to engage in frequent primary care visits, reflecting the need for enhanced follow-up aimed at preventing hospitalization [13]. These encounters may include close monitoring of metabolic parameters, lifestyle support and adjustments in pharmacotherapy. Primary care providers often adopt a proactive approach in these cases, aiming to stabilize glycemic levels and mitigate the risk of complications. Despite these efforts, the persistently high utilization of healthcare resources in this subgroup highlights the challenges of managing complex, multimorbid patients within primary care settings. The findings underscore the substantial burden of cardiovascular disease and CKD in T2D, both of which contribute to increased healthcare utilization. Poor glycemic control, along with reduced eGFR, was associated with more hospitalization and more frequent primary care visits, consistent with prior studies [10,13]. Consequently, elevated HbA1c and lower eGFR were both strong negative predictors, reinforcing the need for early, targeted interventions. Optimizing pharmacotherapy, including SGLT2 inhibitors and GLP-1 receptor agonists may improve glycemic control and slow CKD progression. Despite their proven cardioprotective and renoprotective benefits, these agents remain underutilized, reflecting persistent clinical inertia [33,34].

### Strengths and limitations

This study benefits from a large, geographically defined cohort, enhancing generalizability to similar populations. Comprehensive data from multiple healthcare settings, including hospital admissions, primary care visits and pharmacy records, offers valuable insights into healthcare utilization.

The exclusion of newly diagnosed patients may introduce selection bias, as individuals with a longer disease duration often have different healthcare needs compared to those with a more recent diagnosis. These patients were excluded because they only had diabetes for part of the study year, which would not provide a full-year perspective on healthcare utilization. While this exclusion ensured a more accurate representation of patients with established diabetes, it may limit the applicability of the findings to those recently diagnosed. Newly diagnosed individuals may have distinct healthcare needs, such as more frequent visits for initial management and education, which were not captured in this study. Furthermore, the absence of a wash-out period may introduce bias from individuals with ongoing or chronic hospital use prior to 2020. This limitation should be considered when interpreting associations with hospitalization. Outpatient visits, including emergency department encounters, physician consultations, and nurse or paramedical visits, were recorded as individual events. These were analyzed separately from inpatient care, which was measured in total hospital days.

Another limitation is the use of B-glucose values without differentiation between fasting and non-fasting states. B-glucose values were treated as non-fasting due to the large number and variation in timing. Most tests were performed for monitoring rather than diagnostic purposes, limiting their interpretability. These tests were primarily used to monitor glycemic regulation rather than for diagnostic purposes, which limits their interpretability in terms of metabolic control. The individuals having solely B-glucose may represent a less thoroughly investigated subgroup, introducing a potential selection bias. However, the number was small, limiting the overall impact on results. Furthermore, the lack of data on lifestyle factors, such as diet and physical activity, limits the full understanding of their impact on healthcare utilization. This retrospective observational study identifies associations but cannot establish causality.

## Conclusions

Poor glycemic control and impaired kidney function are associated with increased hospitalization rates in type 2 diabetes. These findings emphasize the need for early intervention and optimized pharmacotherapy, such as SGLT2 inhibitors and GLP-1 receptor agonists, to improve glycemic control and slow CKD progression. Prioritizing regular monitoring and access to guideline-recommended treatments can help reduce hospitalizations and alleviate the healthcare burden.

## Supplementary Material

Supplementary material.docx

Supplementary Figure 1.docx

## Data Availability

The data that support the findings of this study are available on request from the corresponding author. The data are not publicly available due to privacy or ethical restrictions.
