# Supplementary material for: Association of glycemic control and chronic kidney disease with hospitalization in type 2 diabetes in a cross-sectional study in Region Halland
Source: Scand J Prim Health Care. 2025 Dec 1;44(1):1–11. doi: 10.1080/02813432.2025.2591340 (PMC12918358; doi:10.1080/02813432.2025.2591340)
Supplement: Supplementary material.docx [file IPRI_A_2591340_SM3139.docx]

**Supplementary material**

Supplementary Table 1. Diabetes diagnosis and comorbidities used in the study, classified by ICD-10 codes.

| **Diagnose** | **ICD-10 code** |
| --- | --- |
| Diabetes Mellitus | E11-E14 |
| Ischemic Heart Disease | I20-I29 |
| Hypertension | I10 |
| Heart Failure | I50, I42 |
| Peripheral Arterial Disease | I73.9 |
| Cerebrovascular Disease | I63-I69 |
| Chronic Obstructive Pulmonary Disease (COPD) | J44 |
| Diabetic Retinopathy | H36, E113 |
