# Supplementary material for: Association of glycemic control and chronic kidney disease with hospitalization in type 2 diabetes in a cross-sectional study in Region Halland
Source: Scand J Prim Health Care. 2025 Dec 1;44(1):1–11. doi: 10.1080/02813432.2025.2591340 (PMC12918358; doi:10.1080/02813432.2025.2591340)
Supplement: Supplementary Figure 1.docx [file IPRI_A_2591340_SM3138.docx]

Supplementary Figure 1. Flowchart of study population selection for individuals with type 2 diabetes in Region Halland, Sweden.

Total population of Region Halland ~ 330,000 inhabitants

Excluded individuals.

- with new T2D diagnose 2020: 1,028 individuals
- with no data/nonresident 2020: 1,440 individuals
- individuals ≤30 years old: 92 individuals

15,249 Individuals with ICD E11-E14 between 2013-2020

Final cohort 12,689 individuals
